# Supplementary material for: CCCTC-binding factor N-terminal domain regulates clustered protocadherin gene expression by enhancing cohesin processivity[image]
Source: J Biol Chem. 2025 Feb 21;301(4):108337. doi: 10.1016/j.jbc.2025.108337 (PMC11968269; doi:10.1016/j.jbc.2025.108337)
Supplement: Supporting Information [file mmc1.docx]

CCCTC-binding Factor N-terminal Domain

Regulates Clustered Protocadherin Gene

Expression by Enhancing Cohesin Processivity

Yijun Zhang, Qiang Wu*

Center for Comparative Biomedicine, Ministry of Education Key Laboratory of Systems Biomedicine, State Key Laboratory of Medical Genomics, Institute of Systems Biomedicine, Shanghai Jiao Tong University, Shanghai 200240, China

**List of Supporting Materials:**

Figure S1. Generation of models of CTCF ADA mutation.

Figure S2. Genotyping of mouse models of CTCF ADA mutation.

Figure S3. Decreased enrichments of RAD21 at the promoter and enhancer regions of the *cPcdh* genes in *N2a* cells.

Figure S4. Decreased enrichments of RAD21 at the promoter and enhancer regions of the *cPcdh* locus in the mouse brain.

Figure S5. Enrichments of CTCF at the promoter and enhancer regions of the *cPcdh* genes in *N2a* cells.

Figure S6. Enrichments of CTCF at the promoter and enhancer regions of the *cPcdh* locus in the mouse brain.

Figure S7. CTCF YDF motif enables cohesin to have a long residence time on chromatin.

Figure S8. Raw RNA-seq data from the *Wapl* knockdown experiment.

Table S1. Oligonucleotides used in this study.

Table S2. Reagents and plasmids used in this study.

Table S3. *In situ* Hi-C statistics.


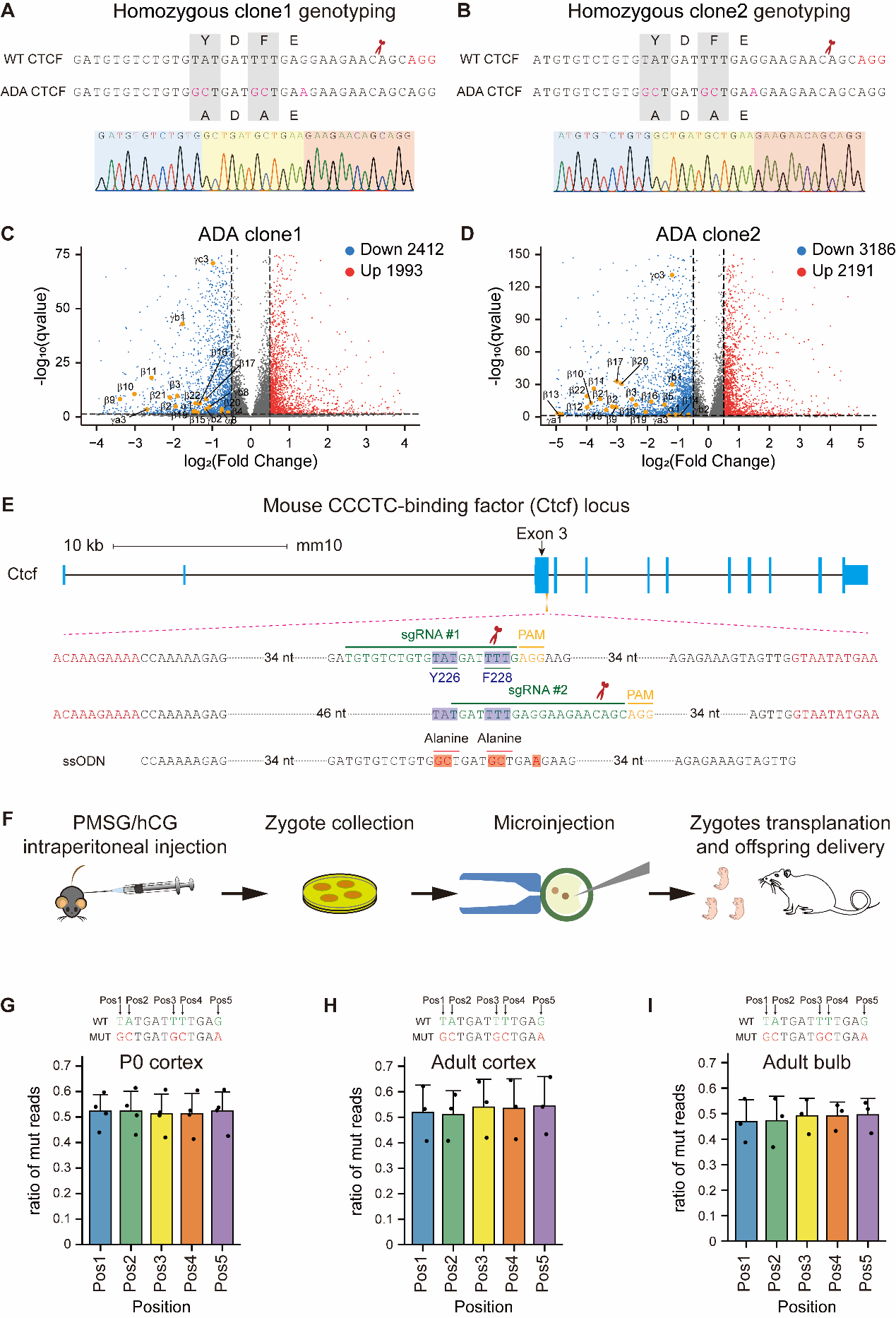


**Figure S1. Generation of models of CTCF ADA mutation.** *A* and *B,* Genotyping of two independent homozygous single-cell clones of CTCF ADA mutation. *C* and *D,* Volcano plots of differentially expressed genes in each of the two independent *N2a* cell clones. The downregulated *cPcdh* genes are marked with yellow dots. *E,* Schematic of CRISPR-mediated DNA editing with a single stranded oligodeoxynucleotide (ssODN) donor for CTCF ADA mutation in mice. *F,* Outline of the procedure for generating CRISPR-mediated site-directed mutagenesis in the N-terminal domain of CTCF in mice. *G*-*I,* Bar plots showing ratios of mutated bases in high-throughput RNA-seq reads from the heterozygous P0 (n=4) (*G*) and adult (n=3) (*H*) cortical, as well as olfactory bulb (n=3) (*I*) tissues. nt, nucleotide. sgRNA, single guide RNA. PAM, protospacer adjacent motif. ssODN, single-stranded oligodeoxynucleotide. PMSG, pregnant mare serum gonadotropin. hCG, human chorionic gonadotropin. Pos, position. Data are mean ± S.D from at least three biological replicates.


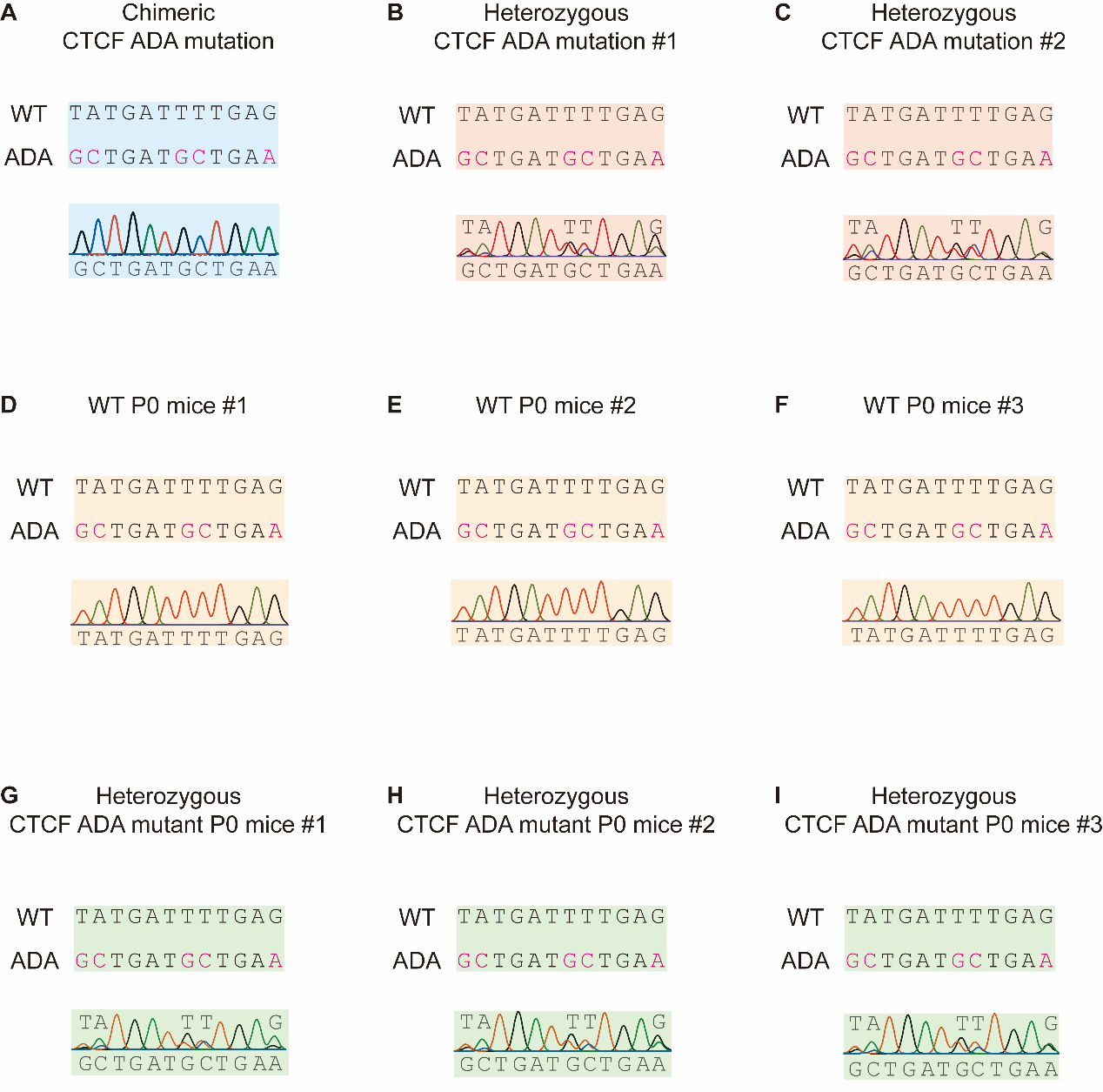


**Figure S2. Genotyping of mouse models of CTCF ADA mutation.** *A,* Genotyping of the chimeric CTCF ADA mouse by TA cloning and Sanger sequencing. *B* and *C,* Genotyping of the heterozygous CTCF ADA mice by Sanger sequencing. *D*-*I,* Genotyping of a litter of three P0 wild-type mice and three P0 heterozygous CTCF ADA mice by Sanger sequencing.


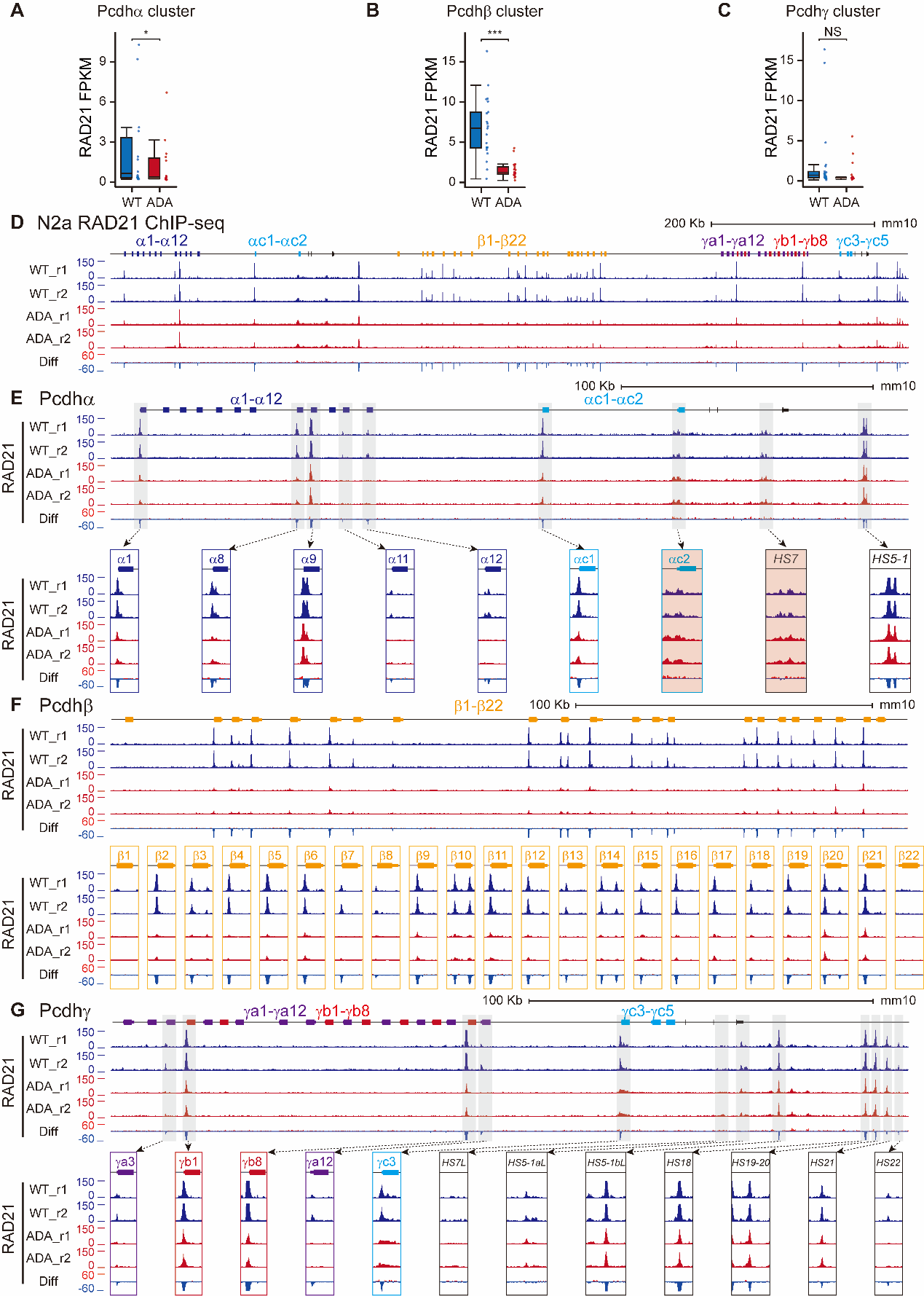


**Figure S3. Decreased enrichments of RAD21 at the promoter and enhancer regions of the *cPcdh* genes in *N2a* cells.** *A*-*C,* Quantification of alterations of RAD21 enrichments at the *Pcdh* *α* (*A*), *β* (*B*), and *γ* (*C*) clusters in the cellular model. Statistical significance of changes of RAD21 enrichment was computed using the R package rstatix (v0.7.0), with significance levels denoted as following: ‘*’ for *P* ≤ 0.05, ‘**’ for *P* ≤ 0.01, and ‘***’ for *P* ≤ 0.001, using paired two-tailed Student’s *t*-test. *D,* RAD21 ChIP-seq profiles at the *cPcdh* locus in CTCF ADA *N2a* clones compared to the WT clone. *E*-*G,* Close-up of RAD21 profiles at the *Pcdh* *α* (*E*), *β* (*F*), and *γ* (*G*) clusters showing decreased enrichments of RAD21 at *cPcdh* variable exons and enhancers. Note that as internal controls with no CTCF site, there do not appear decreased RAD21 enrichments at *αc2* and *HS7*. r1, replicate 1. r2, replicate 2.


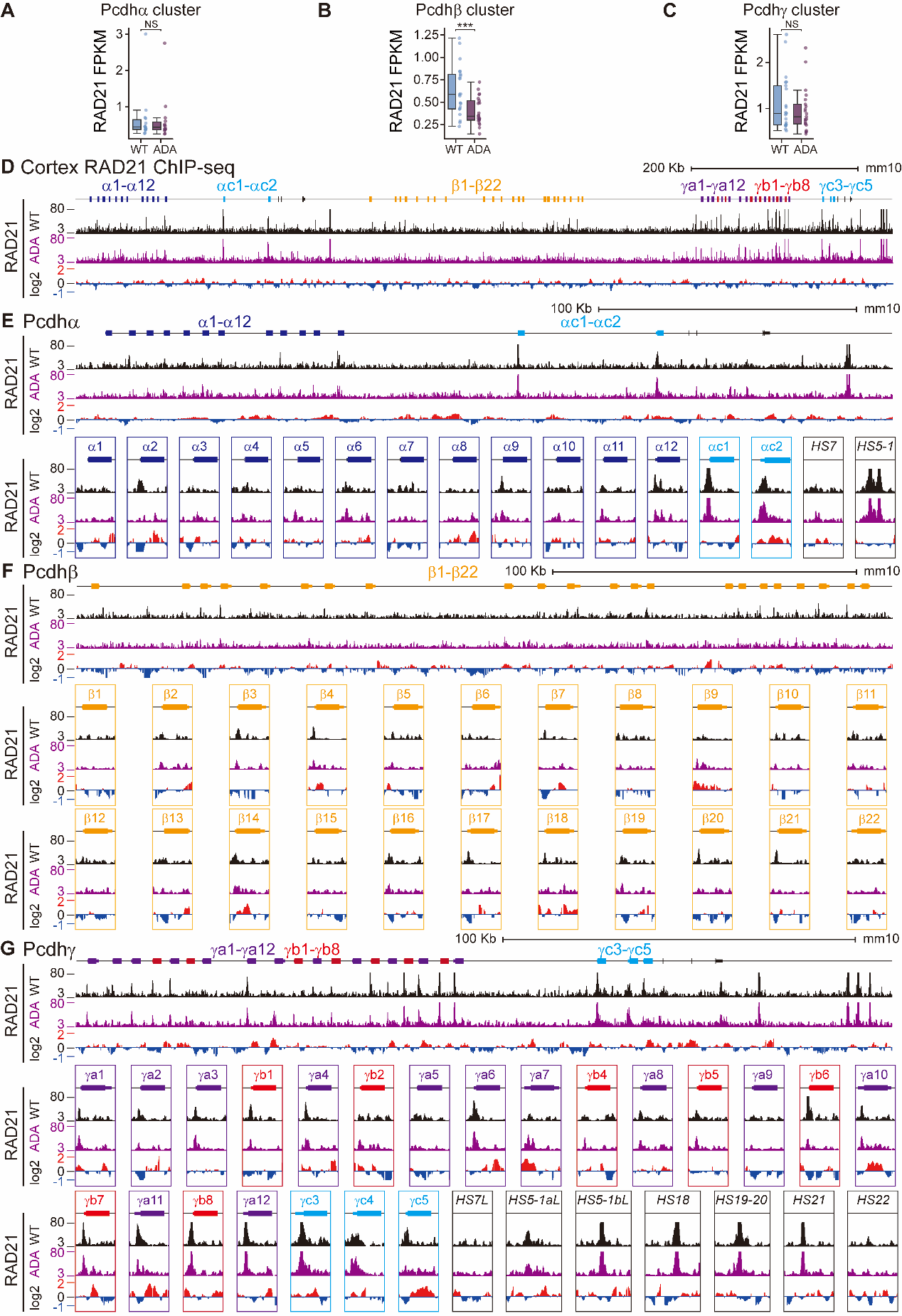


**Figure S4. Decreased enrichments of RAD21 at the promoter and enhancer regions of the *cPcdh* locus in the mouse brain.** *A*-*C,* Quantification of alterations of RAD21 enrichments at the *Pcdh* *α* (*A*), *β* (*B*), and *γ* (*C*) clusters in the mouse model. Statistical significance of alterations of RAD21 enrichment was calculated using paired two-tailed Student’s *t*-test, with significance levels denoted as following: ‘*’ for *P* ≤ 0.05, ‘**’ for *P* ≤ 0.01, and ‘***’ for *P* ≤ 0.001. *D,* RAD21 ChIP-seq profiles at the *cPcdh* locus in heterozygous CTCF ADA mice compared to WT littermates. *E*-*G,* Close-up of RAD21 profiles at the *Pcdh* *α* (*E*), *β* (*F*), and *γ* (*G*) clusters showing decreased enrichments of RAD21 at *cPcdh* variable exons and enhancers.


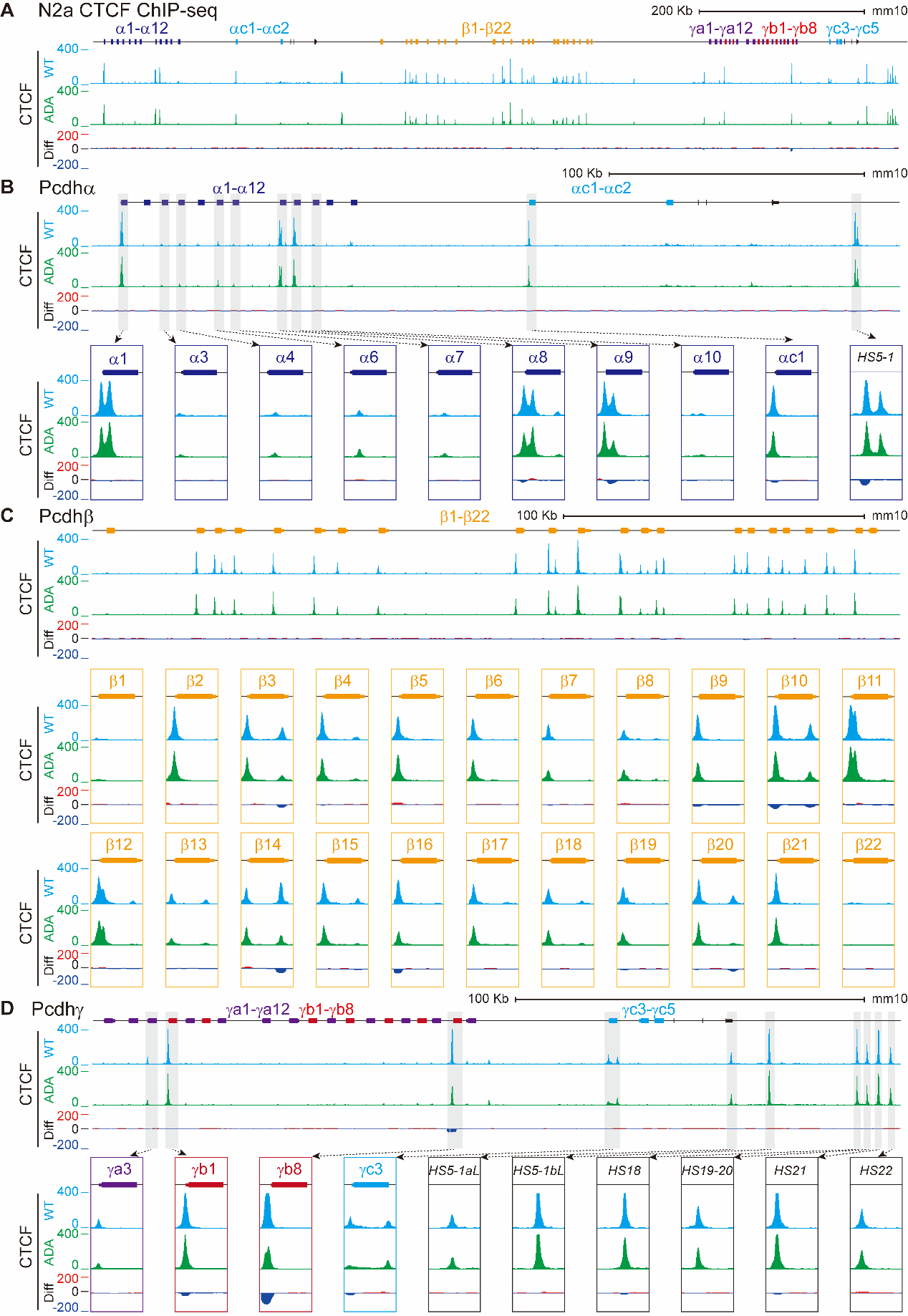


**Figure S5. Enrichments of CTCF at the promoter and enhancer regions of the *cPcdh* genes in *N2a* cells.** *A,* CTCF ChIP-seq profiles at the *cPcdh* locus in CTCF ADA *N2a* clones compared to the WT clone. *B*-*D,* Close-up of CTCF profiles at the *Pcdh* *α* (*B*), *β* (*C*), and *γ* (*D*) clusters showing no decrease of CTCF enrichments at *cPcdh* variable exons and enhancers.


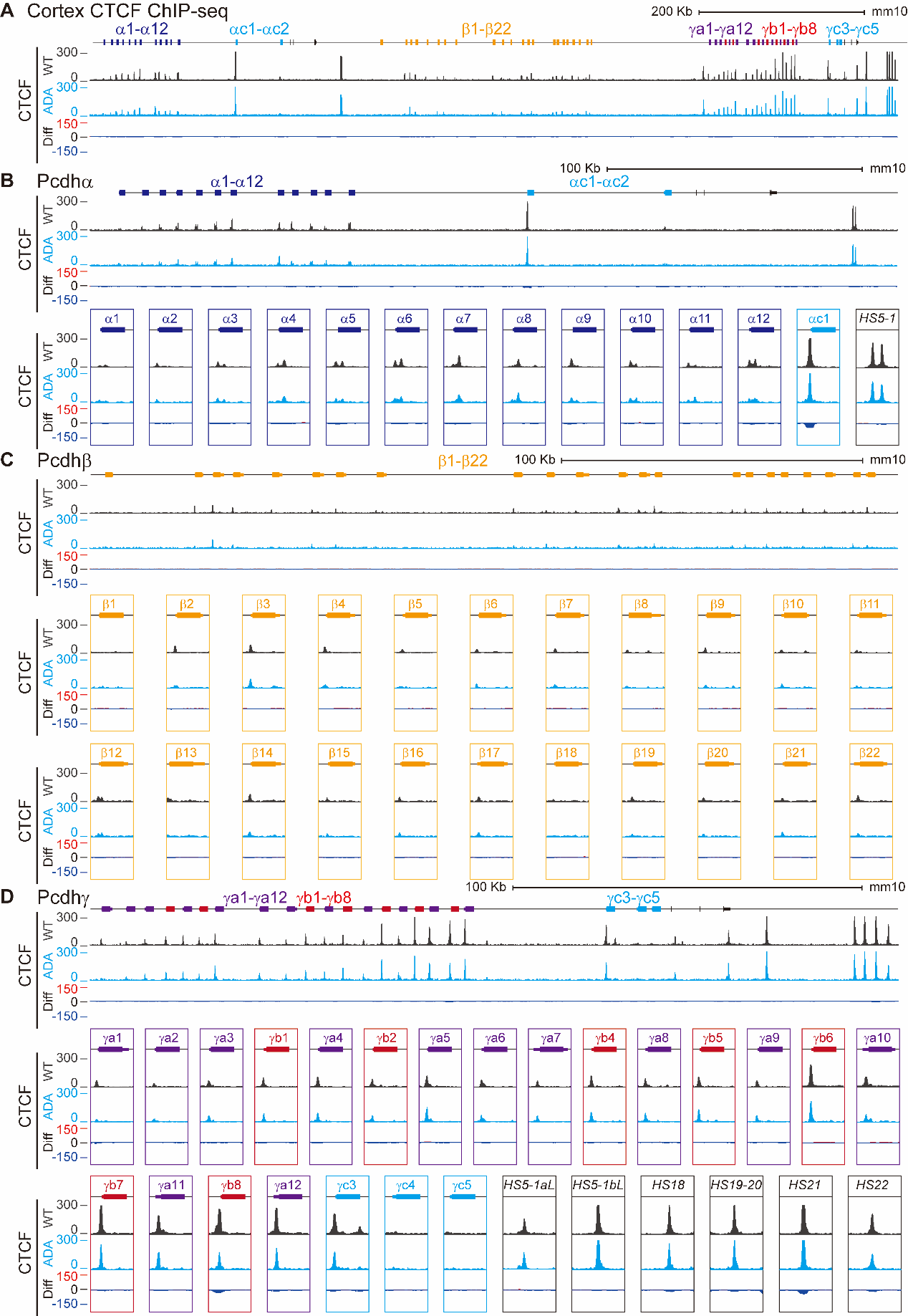


**Figure S6. Enrichments of CTCF at the promoter and enhancer regions of the c*Pcdh* locus in the mouse brain.** *A,* ChIP-seq profiles of CTCF at the *cPcdh* locus in WT and heterozygous CTCF ADA mice. *B*-*D,* Close-up of CTCF profiles at the *Pcdh* *α* (*B*), *β* (*C*), and *γ* (*D*) clusters.


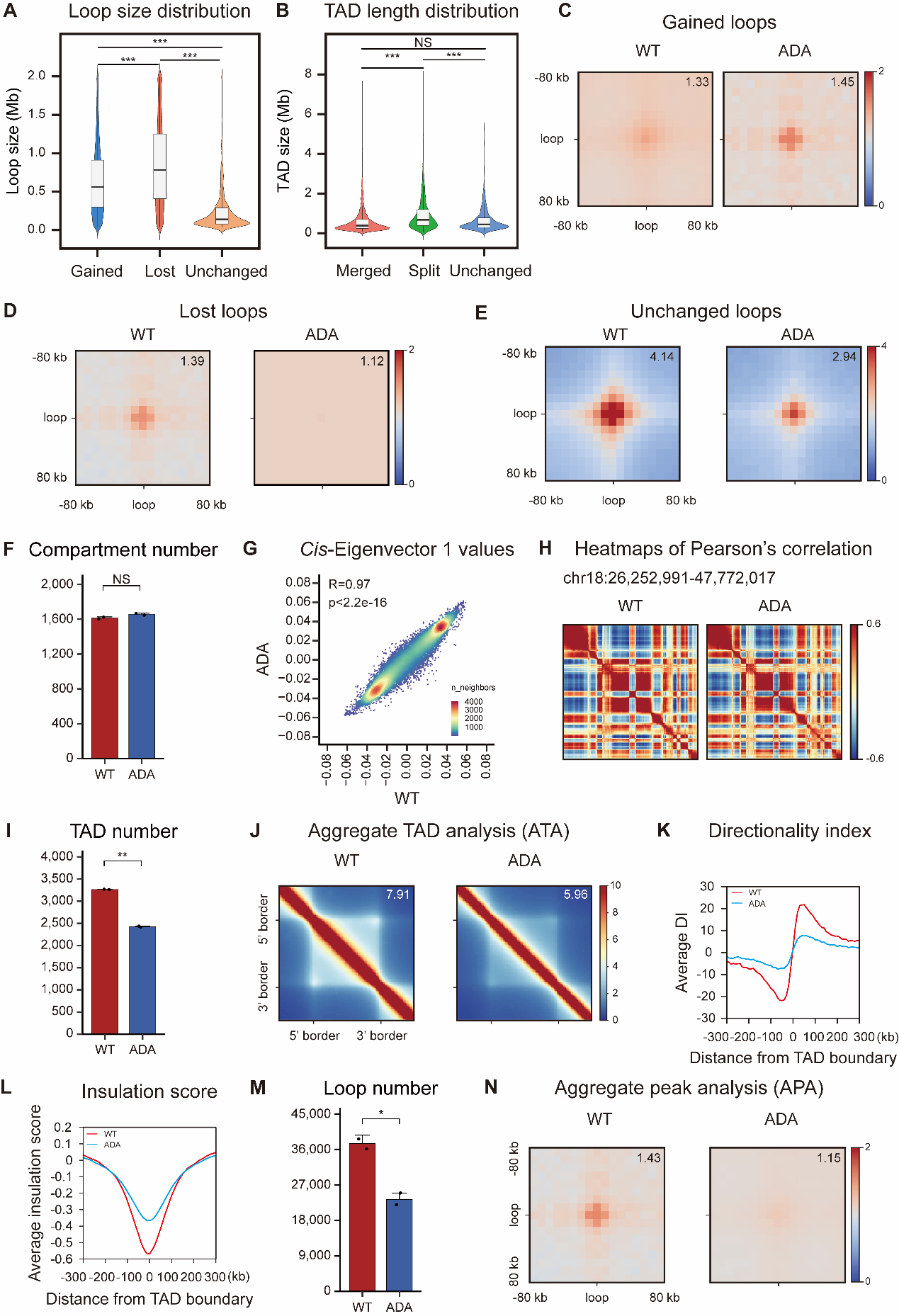


**Figure S7. CTCF YDF motif enables cohesin to have a long residence time on chromatin.** *A,* Violin plot showing the distribution of loop sizes in gained, lost, and unchanged regions, showing that unchanged loops display the smallest size, whereas lost loops typically tend to have the largest size, and gained loops are in an intermediate range. *B,* Violin plot depicting the distribution of lengths of merged, split, and unchanged TADs, showing that the length of split TADs tends to be significant longer than that of the merged or unchanged TADs. Statistical significance of alterations was calculated using unpaired two-tailed Student’s *t*-test, with significance levels denoted as following: ‘*’ for *P* ≤ 0.05, ‘**’ for *P* ≤ 0.01, and ‘***’ for *P* ≤ 0.001. *C*-*E,* Aggregate peak analysis (APA) of signals of the gained (*C*), lost (*D*), or unchanged (*E*) loops. The APA analysis revealed a significant decrease in loop strength of lost loops, alongside a notable increase in looping strength based on the gained loops upon CTCF ADA mutation. The unchanged loops also exhibited a significant decrease in looping strength, indicating that CTCF ADA mutation destabilizes the extruding cohesin complex on chromatin. In each panel, the APA value is indicated in the upper-right corner. *F,* Bar plot showing numbers of compartment in wild-type and heterozygous CTCF ADA mice (n=2). Data are mean ± S.D from two biological replicates, **P* < 0.05, ***P* < 0.01, ****P* < 0.001; unpaired two-tailed Student’s *t*-test. *G,* Scatterplot displaying the Pearson’s correlation of *cis*-eigenvector 1 values between wild-type and heterozygous CTCF ADA mice. *H,* Heatmaps of Pearson’s correlation at chr18: 26,252,991-47,772,017 in wild-type and heterozygous CTCF ADA mice, showing an unchanged compartmentalization upon CTCF ADA mutation. *I,* Numbers of TADs in wild-type and heterozygous CTCF ADA mice (n=2). Data are mean ± S.D from two biological replicates, **P* < 0.05, ***P* < 0.01, ****P* < 0.001; unpaired two-tailed Student’s *t*-test. *J,* Aggregate TAD analysis (ATA) in wild-type and heterozygous CTCF ADA mice, showing a significant decrease of intra-TAD contacts. *K,* Pileup of the directionality index scores in 300-Kb regions centered on all TAD boundaries in wild-type and heterozygous CTCF ADA mice, showing the weakened insulation at TAD boundaries. DI, directionality index. *L,* Averaged insulation scores in 300-Kb regions centered on all TAD boundaries in wild-type and heterozygous CTCF ADA mice. *M,* Hi-C loop number in wild-type and heterozygous CTCF ADA mice (n=2). Data are mean ± S.D from two biological replicates, **P* < 0.05, ***P* < 0.01, ****P* < 0.001; unpaired two-tailed Student’s *t*-test. *N,* Aggregate peak analysis (APA) depicting the superimposed signal for genome-wide loops estimated from Hi-C data of wild-type and heterozygous CTCF ADA mice, showing global weakening of chromatin loops upon CTCF ADA mutation. In each panel, the APA value is indicated in the upper-right corner.

**
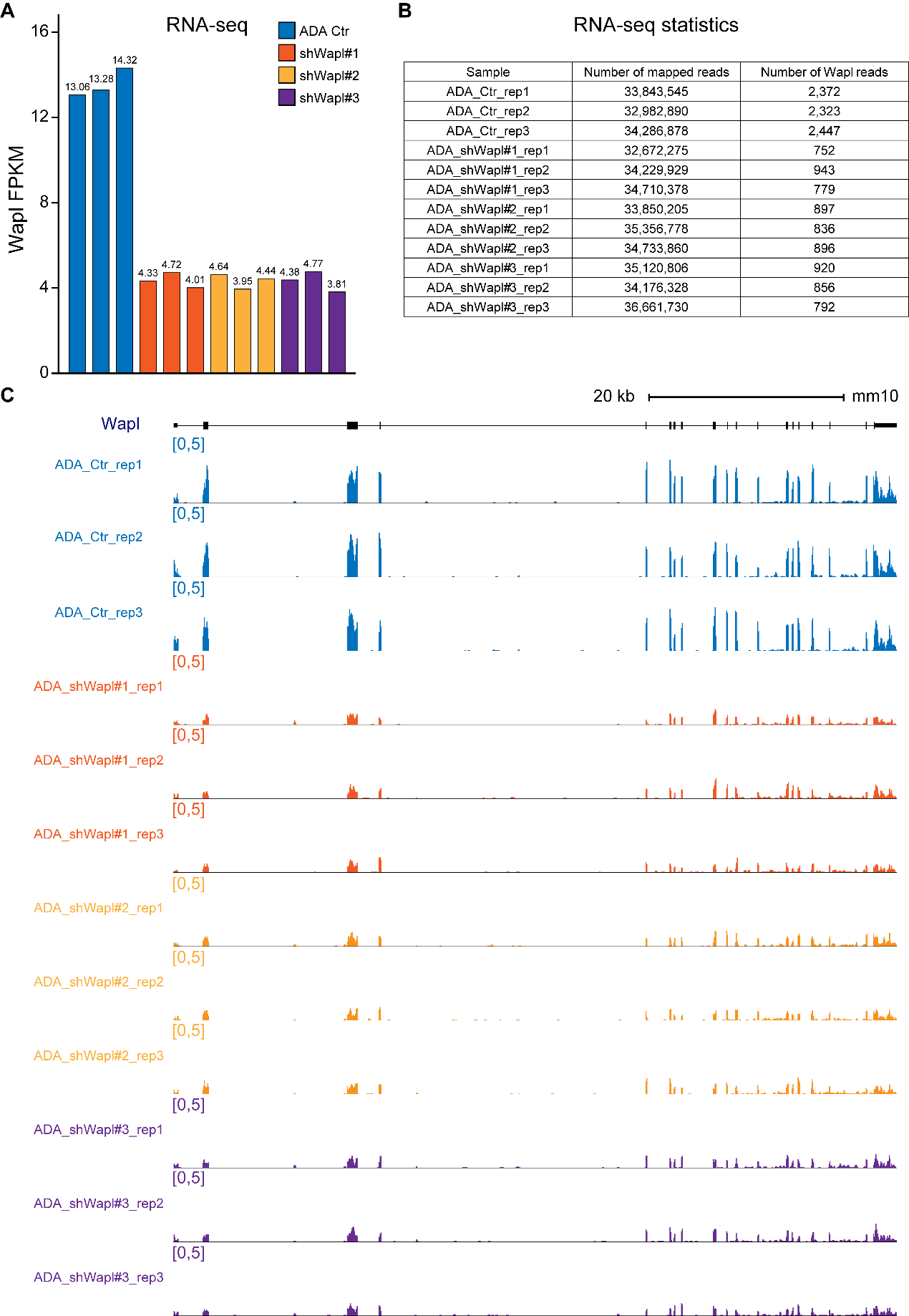
**

**Figure S8. Raw RNA-seq data from the *Wapl* knockdown experiment.**

**Table S1. Oligonucleotides used in this study**

| **Oligonucleotides for the construction of plasmids for transfection** | |
| --- | --- |
| Mut_sgRNA_F | ACCGTGATTTTGAGGAAGAACAGC |
| Mut_sgRNA_R | AAACGCTGTTCTTCCTCAAAATCA |
| Left_Mut_F | AAAACGACGGCCAGTGAATTCGGTTCTTAGAGATAGGGTTTCTCTGT |
| Left_Mut_R | TTCAGCATCAGCCACAGACACATCCACGTCTTTG |
| Right_Mut_F | TGTCTGTGGCTGATGCTGAAGAAGAACAGCAGGAAGGACTGC |
| Right_Mut_R | GACCATGATTACGCCAAGCTTTTGCTGGCTTAACTAGGTGAG |
| **Oligonucleotides for the sgRNA generation for microinjection** | |
| T7_Mut-sgRNA-F1 | taatacgactcactataGGTGTCTGTGTATGATTTTGgttttagagctagaaatag |
| T7_Mut-sgRNA-F2 | taatacgactcactataggTGATTTTGAGGAAGAACAGCgttttagagctagaaatag |
| sgRNA-R | AAAAGCACCGACTCGGTGCC |
| ssODN_Mut | CCAAAAAGAGCAAACTTCGTTACACAGAAGAGGGCAAAGACGTGGATGTGTCTGTGGCTGATGCTGAAGAAGAACAGCAGGAAGGACTGCTGTCTGAGGTTAATGCAGAGAAAGTAGTTG |
| **Oligonucleotides for the cell clone genotyping** | |
| geno_F1_Wt | GGATGTGTCTGTGTATGATTTTGAG |
| geno_F1_Mut | TGTCTGTGGCTGATGCTGAA |
| geno_R1 | CCAGGGCTATGTAGAAGACTCTG |
| **Oligonucleotides for amplifying targeted sites for the mouse genotyping via Sanger sequencing** | |
| geno_226228_F | GGAGAAGTGGAGACACTAGAGCAG |
| geno_226228_R | TAACTTGCATAGAGGCTGTTGTATC |
| **Oligonucleotides for final construction of library for the high-throughput sequencing** | |
| Adapter-HTS_F | 5’-ACACTCTTTCCCTACACGACGCTCTTCCGATC*T-3’ |
| Adapter-HTS_R | 5’-p-GATCGGAAGAGCACACGTCTGAACTCCAGTC-3’ |
| P5 index-1 | AATGATACGGCGACCACCGAGATCTACACTATAGCCTACACTCTTTCCCTACACGACGCTCTTCCGATCT |
| P5 index-2 | AATGATACGGCGACCACCGAGATCTACACATAGAGGCACACTCTTTCCCTACACGACGCTCTTCCGATCT |
| P5 index-3 | AATGATACGGCGACCACCGAGATCTACACCCTATCCTACACTCTTTCCCTACACGACGCTCTTCCGATCT |
| P5 index-4 | AATGATACGGCGACCACCGAGATCTACACGGCTCTGAACACTCTTTCCCTACACGACGCTCTTCCGATCT |
| P5 index-5 | AATGATACGGCGACCACCGAGATCTACACAGGCGAAGACACTCTTTCCCTACACGACGCTCTTCCGATCT |
| P5 index-6 | AATGATACGGCGACCACCGAGATCTACACTAATCTTAACACTCTTTCCCTACACGACGCTCTTCCGATCT |
| P5 index-7 | AATGATACGGCGACCACCGAGATCTACACCAGGACGTACACTCTTTCCCTACACGACGCTCTTCCGATCT |
| P5 index-8 | AATGATACGGCGACCACCGAGATCTACACGTACTGACACACTCTTTCCCTACACGACGCTCTTCCGATCT |
| P5 index-9 | AATGATACGGCGACCACCGAGATCTACACGACCTGTAACACTCTTTCCCTACACGACGCTCTTCCGATCT |
| P5 index-10 | AATGATACGGCGACCACCGAGATCTACACATGTAACTACACTCTTTCCCTACACGACGCTCTTCCGATCT |
| P5 index-11 | AATGATACGGCGACCACCGAGATCTACACGTTTCAGAACACTCTTTCCCTACACGACGCTCTTCCGATCT |
| P5 index-12 | AATGATACGGCGACCACCGAGATCTACACCACAGGATACACTCTTTCCCTACACGACGCTCTTCCGATCT |
| P7-index-1 | CAAGCAGAAGACGGCATACGAGATCGAGTAATGTGACTGGAGTTCAGACGTGTGCTCTTCCGATCT |
| P7-index-2 | CAAGCAGAAGACGGCATACGAGATTCTCCGGAGTGACTGGAGTTCAGACGTGTGCTCTTCCGATCT |
| P7-index-3 | CAAGCAGAAGACGGCATACGAGATAATGAGCGGTGACTGGAGTTCAGACGTGTGCTCTTCCGATCT |
| P7-index-4 | CAAGCAGAAGACGGCATACGAGATGGAATCTCGTGACTGGAGTTCAGACGTGTGCTCTTCCGATCT |
| P7-index-5 | CAAGCAGAAGACGGCATACGAGATTTCTGAATGTGACTGGAGTTCAGACGTGTGCTCTTCCGATCT |
| P7-index-6 | CAAGCAGAAGACGGCATACGAGATACGAATTCGTGACTGGAGTTCAGACGTGTGCTCTTCCGATCT |
| P7-index-7 | CAAGCAGAAGACGGCATACGAGATAGCTTCAGGTGACTGGAGTTCAGACGTGTGCTCTTCCGATCT |
| P7-index-8 | CAAGCAGAAGACGGCATACGAGATGCGCATTAGTGACTGGAGTTCAGACGTGTGCTCTTCCGATCT |
| P7-index-9 | CAAGCAGAAGACGGCATACGAGATCATAGCCGGTGACTGGAGTTCAGACGTGTGCTCTTCCGATCT |
| P7-index-10 | CAAGCAGAAGACGGCATACGAGATTTCGCGGAGTGACTGGAGTTCAGACGTGTGCTCTTCCGATCT |
| P7-index-11 | CAAGCAGAAGACGGCATACGAGATGCGCGAGAGTGACTGGAGTTCAGACGTGTGCTCTTCCGATCT |
| P7-index-12 | CAAGCAGAAGACGGCATACGAGATCTATCGCTGTGACTGGAGTTCAGACGTGTGCTCTTCCGATCT |
| **Oligonucleotides for QHR-4C experiments** | |
| Adapter-4C_F | 5’-GACGTGTGCTCTTCCGATCTGNNNNNN-3’NH2 C6 |
| Adapter-4C_R | 5’-p-CAGATCGGAAGAGCACACGTC-3’NH2 C6 |
| biotin_mHS5-1 | 5’-biotin-GTGGCTTTGTTACTCTAGGAACAG |
| biotin_mSEbc | 5’-biotin-AACTATGCTCAGGGCCTCTGG |
| biotin_mSEd | 5’-biotin-TCCCTGCCAGCTGAGCTAAG |
| biotin_mSEe | 5’-biotin-TTAGAGGACACGCTGCCGTC |
| qhr-mhs51-F | AATGATACGGCGACCACCGAGATCTACACTATAGCCTACACTCTTTCCCTACACGACGCTCTTCCGATCTGGAGGTTAAAGCAAAGACTAAGATC |
| qhr-m-se-bc-F | AATGATACGGCGACCACCGAGATCTACACATAGAGGCACACTCTTTCCCTACACGACGCTCTTCCGATCTGAGGCTTATGGGCCTTACAGATC |
| qhr-m-se-d-F | AATGATACGGCGACCACCGAGATCTACACCCTATCCTACACTCTTTCCCTACACGACGCTCTTCCGATCTACAGCAGGCAGCAGCTGATC |
| qhr-m-se-e-F | AATGATACGGCGACCACCGAGATCTACACGGCTCTGAACACTCTTTCCCTACACGACGCTCTTCCGATCTCCAGATTCTGAACCCGACAGATC |
| **Oligonucleotides for constructing plasmids for Wapl knockdown** | |
| shCtr_F | CCGGCAACAAGATGAAGAGCACCAACTCGAGTTGGTGCTCTTCATCTTGTTGTTTTTG |
| shCtr_R | AATTCAAAAACAACAAGATGAAGAGCACCAACTCGAGTTGGTGCTCTTCATCTTGTTG |
| shWapl_1F | CCGGGCAGTGGCTTCTGAATATAAACTCGAGTTTATATTCAGAAGCCACTGCTTTTTG |
| shWapl_1R | AATTCAAAAAGCAGTGGCTTCTGAATATAAACTCGAGTTTATATTCAGAAGCCACTGC |
| shWapl_2F | CCGGGCCCAATTTCAAACCAGATATCTCGAGATATCTGGTTTGAAATTGGGCTTTTTG |
| shWapl_2R | AATTCAAAAAGCCCAATTTCAAACCAGATATCTCGAGATATCTGGTTTGAAATTGGGC |
| shWapl_3F | CCGGAACGGACTACTCTTAGTACAACTCGAGTTGTACTAAGAGTAGTCCGTTTTTTTG |
| shWapl_3R | AATTCAAAAAAACGGACTACTCTTAGTACAACTCGAGTTGTACTAAGAGTAGTCCGTT |
| **Oligonucleotides for constructing plasmids for CTCF overexpression** | |
| mCtcf_EcoRI_F | CCGGAATTCATGGAAGGTGAGGCGGTTGA |
| mCtcf_NotI_R | AAGGAAAAAAGCGGCCGCCCGGTCCATCATGCTGAGGA |
| CtcfOE_genoF1 | ATGGAAGGTGAGGCGGTTGA |
| CtcfOE_genoF2 | CACTGATGAGAGACCACACAAATGC |
| CtcfOE_genoF3 | GTGATTATGCTTGTAGACAGGAGCG |

**Table S2. Reagents and plasmids used in this study**

| **Antibodies** | | |
| --- | --- | --- |
| Anti-CTCF Antibody | Millipore | Cat# 07-729 |
| Anti-Rad21 Antibody | Abcam | Cat# ab992 |
| Anti-V5 antibody | proteintech | Cat# 14440-1-AP |
| Anti-β-actin antibody | proteintech | Cat# 66009-1-Ig |
| IRDye680-conjugated goat anti-rabbit secondary antibody | Biosciences | Cat# 926-68070 |
| IRDye800-conjugated goat anti-mouse secondary antibody | Biosciences | Cat# 926-32213 |
| **Chemicals** | | |
| MEM | Gibco | Cat# 11095080 |
| MEM-NEAA | Gibco | Cat# 11140050 |
| Penicillin-streptomycin | Gibco | Cat# 15140122 |
| Fetal Bovine Serum | Sigma-Aldrich | Cat# F0193 |
| PBS | Gibco | Cat# 70011044 |
| 0.25% trypsin-EDTA | Gibco | Cat# 25200056 |
| *Bsa*I | NEB | Cat# R3733S |
| *Eco*RI | NEB | Cat# R3101S |
| *Hin*dIII | NEB | Cat# R3104S |
| *Xba*I | NEB | Cat# R0145S |
| *Mbo*I | NEB | Cat# R0147L |
| *Dpn*II | NEB | Cat# R0543L |
| *Age*I | NEB | Cat# R3552S |
| *Not*I | NEB | Cat# R3189S |
| Lipofectamine 3000 reagents | Invitrogen | Cat# L3000015 |
| Puromycin dihydrochloride | Solarbio | Cat# P8230-25mg |
| Phanta Max Super-Fidelity DNA Polymerase | Vazyme | Cat# P505-d1 |
| RNase-free water | Invitrogen | Cat# 10977023 |
| Phenol-chloroform | ACMEC | Cat# AC13309 |
| MEGAshortscript Kit | Invitrogen | Cat# AM1354 |
| mMACHINE T7 ULTRA kit | Invitrogen | Cat# AM1345 |
| MEGAclear Kit | Invitrogen | Cat# AM1908 |
| Sodium acetate | Invitrogen | Cat# AM9740 |
| Glycogen | Thermo Scientific | Cat# R0551 |
| PMSG | Solarbio | Cat# P9970 |
| hCG | MCE | Cat# HY-107953 |
| Sodium Chloride | Sigma-Aldrich | Cat# S3014 |
| Hyaluronidase | Sigma-Aldrich | Cat# H4272-30MG |
| M2 medium | Sigma-Aldrich | Cat# MR-015 |
| Mineral oil | Sigma-Aldrich | Cat# M5310 |
| TRIzol Reagent | Invitrogen | Cat# 15596018 |
| AMPure XP beads | Beckman | Cat# A63881 |
| 16% Formaldehyde | Thermo Scientific | Cat# 28906 |
| Glycine | Invitrogen | Cat# 15527013 |
| Sodium deoxycholate | Sigma-Aldrich | Cat# 30970 |
| SDS | Sigma-Aldrich | Cat# 71736 |
| Triton X-100 | Sigma-Aldrich | Cat# T8787 |
| EDTA | Sigma-Aldrich | Cat# E1644 |
| LiCl | Sigma-Aldrich | Cat# L4408 |
| NP40 | Sigma-Aldrich | Cat# I8896 |
| NeuroBasal medium | Gibco | Cat# 10888022 |
| EDTA-free protease inhibitor | Roche | Cat# 04693116001 |
| Protein A-agarose beads | Millipore | Cat# 16-157 |
| RNase A | Thermo Scientific | Cat# EN0531 |
| Proteinase K | Invitrogen | Cat# AM2546 |
| Equalbit 1 × dsDNA HS Assay Kit | Vazyme | Cat# EQ121-01 |
| DNA Polymerase I, Large (Klenow) Fragment | NEB | Cat# M0210S |
| Biotin-14-dATP | Thermo Scientific | Cat# 19524016 |
| Deoxynucleotide (dNTP) Solution Set | NEB | Cat# N0446S |
| T4 DNA ligase | NEB | Cat# M0202S |
| Streptavidin magnetic C1 beads | Invitrogen | Cat# 65001 |
| Dynabeads M-280 | Thermo Scientific | Cat# 11206D |
| **Commercial assays** | | |
| ClonExpress MultiS One Step Cloning Kit | Vazyme | Cat# C113 |
| VAHTS Universal RNA-seq Library Prep Kit for Illumina | Vazyme | Cat# NR604 |
| VAHTS Universal DNA Library Prep Kit for Illumina | Vazyme | Cat# ND607 |
| QIAquick PCR Purification Kit | Qiagen | Cat# 28104 |
| MinElute Gel Extraction Kit | Qiagen | Cat# 28604 |
| High-Pure PCR Product Purification kit | Roche | Cat# 11732676001 |
| BCA protein assay kit | Beyotime | Cat# P0009 |
| **Plasmids** | | |
| pGL3-U6-sgRNA-vector | This study | N/A |
| pcDNA3.1-Cas9-vector | This study | N/A |
| pUC19_CtcfMut-vector | This study | N/A |
| pLKO.1-shCtr-vector | This study | N/A |
| pLKO.1-shWapl-vector#1 | This study | N/A |
| pLKO.1-shWapl-vector#2 | This study | N/A |
| pLKO.1-shWapl-vector#3 | This study | N/A |
| pMD2.G-vector | Addgene | Cat# 12259 |
| psPAX2-vector | Addgene | Cat# 12260 |
| pLVX-IRES-Puro | This study | N/A |
| pLVX-mCTCF-V5 | This study | N/A |
| **Experimental models: Cell lines** | | |
| Mouse: Neuro-2a | ATCC | Cat# CCL-131 |
| Human: HEK293T | ATCC | Cat# CRL-3216 |

**Table S3. *In situ* Hi-C statistics**

|  | WT_rep1 | WT_rep2 | ADA_rep1 | ADA_rep2 |
| --- | --- | --- | --- | --- |
| Valid_interaction_pairs | 1,169,250,659 | 1,282,103,567 | 1,056,315,746 | 1,164,695,035 |
| Valid_interaction_pairs_FF | 289,994,816 | 318,174,399 | 263,315,179 | 289,472,201 |
| Valid_interaction_pairs_RR | 289,921,136 | 318,100,330 | 263,312,165 | 289,512,479 |
| Valid_interaction_pairs_RF | 288,683,416 | 316,540,127 | 262,692,179 | 288,544,162 |
| Valid_interaction_pairs_FR | 300,651,291 | 329,288,711 | 266,996,223 | 297,166,193 |
| Dangling_end_pairs | 66,522,829 | 75,925,553 | 46,541,563 | 76,450,325 |
| Religation_pairs | 33,099,080 | 36,340,974 | 16,334,596 | 27,451,754 |
| Self_Cycle_pairs | 3,555,453 | 3,745,368 | 2,968,630 | 2,581,732 |
| Single-end_pairs | 0 | 0 | 0 | 0 |
| Filtered_pairs | 0 | 0 | 0 | 0 |
| Dumped_pairs | 467,118 | 397,900 | 413,642 | 261,100 |
| Valid_interaction | 1,169,250,659 | 1,282,103,567 | 1,056,315,746 | 1,164,695,035 |
| Valid_interaction_rmdup | 1,075,932,537 | 1,176,201,570 | 869,554,809 | 1,019,127,664 |
| Trans_interaction | 373,187,735 | 395,027,651 | 402,791,776 | 490,664,581 |
| Cis_interaction | 702,744,802 | 781,173,919 | 466,763,033 | 528,463,083 |
| Cis_shortRange | 153,890,242 | 171,454,035 | 82,565,133 | 99,359,414 |
| Cis_longRange | 548,854,560 | 609,719,884 | 384,197,900 | 429,103,669 |
